# Diagnostic Drama. Use of ICDAS II and Fluorescence-Based Intraoral Camera in Early Occlusal Caries Detection: A Clinical Study

**DOI:** 10.3390/ijerph17082937

**Published:** 2020-04-24

**Authors:** Marta Mazur, Maciej Jedliński, Artnora Ndokaj, Denise Corridore, Antonello Maruotti, Livia Ottolenghi, Fabrizio Guerra

**Affiliations:** 1 Department of Oral and Maxillofacial Surgery, Sapienza University of Rome, 00185 Rome, Italy; artnora.ndokaj@uniroma1.it (A.N.); denise.corridore@uniroma1.it (D.C.); livia.ottolenghi@uniroma1.it (L.O.); fabrizio.guerra@uniroma1.it (F.G.); 2 Department of Conservative Dentistry and Endodontics, Pomeranian Medical University in Szczecin, 70-204 Szczecin, Poland; maciej.jedlinski@hotmail.com; 3 Department of Mathematics, University of Bergen, 5020 Bergen, Norway; Antonello.Maruotti@uib.no

**Keywords:** occlusal caries, dentine caries, caries detection, caries diagnostics, fluorescence camera, VistaCam, ICDAS-II

## Abstract

Background: Early diagnosis of occlusal caries is of paramount importance for a minimally invasive approach in dentistry. The aim of the present in vivo clinical prospective study was to compare the diagnostic outcomes of visual subjective evaluation between the International Caries Detection and Assessment System (ICDAS-II) and an intraoral fluorescence-based camera (VistaCam iX Proof, Dürr Dental, Bietigheim-Bissingen, Germany) for the detection of pits and fissures in early caries lesions of posterior teeth. Methods: The study included 1011 posterior teeth in 255 patients aged 13–20 years (mean age 16 ± 2.2 years). Two blinded operators evaluated all the occlusal surfaces and the first assigned an ICDAS-II code, while the second assessed the VistaCam score: sound enamel (score 0–1.2); initial enamel decay (score 1.2–1.5); dentine caries (score 1.5–3). Results: Some 283 (28%) of the assessed teeth were ICDAS-II code 0; 334 (33%) code 1; 189 (18.7%) code 2; 176 (17.4%) code 3; and 29 (2.9%) code 4. The level of agreement between the two procedures was expressed by using Cohen’s and Fleiss’ kappa statistics and performing McNemar’s test. VistaCam assessed in 513 (50.7%) sound enamel; in 292 (28.9%) initial enamel decay; and in 206 (20.4%) dentine caries. Conclusions: This comparative study showed a poor agreement between the two diagnostic methods, especially between ICDAS-II 0, 1 and 2 codes and fluorescence assessments.

## 1. Introduction

One of the most prominent trends in modern dentistry is minimally invasive dentistry (MID), which aims to preserve as much hard tooth tissue as possible and to prevent the future loss of teeth [1]. However, to incorporate this concept into clinical practice, it is crucial to diagnose dental caries as early and as accurately as possible.

The visual-tactile evaluation is usually carried out in accordance with the International Caries Detection and Assessment System (ICDAS-II), which guarantees standardization and comparability for proper diagnosis, data collection and future re-evaluation [2]. However, visual evaluation can underestimate occlusal caries [3]. To maximize the diagnostic efficacy of visual evaluation, additional diagnostic tools can be used, such as fluorescence-based cameras. This technology allows the assessment of enamel demineralization from its very first stages, as well as enables the monitoring of its development over time. Moreover, this equipment has been shown to be accurate and sensitive, and does not expose the patient to the potential health risk of ionizing radiation [4].

Many trials have been published on the accuracy and efficacy of each of the above-mentioned diagnostic methods, but scant data are available on the comparative diagnostic outcomes on early occlusal caries assessment [5,6,7]. Therefore, the aim of this in vivo study was to compare the performance of both ICDAS-II and intraoral fluorescence camera for the detection of pits and fissures in early caries lesions. The null hypothesis is that the two methods would have the same performance.

## 2. Materials and Methods

This in vivo prospective study was conducted at the 1st Observation Unit of Oral and Maxillofacial Sciences Department, “Sapienza” University of Rome. The study was approved by the Local Ethics Committee (*n*. 4280). All patients signed informed consent forms. All the procedures were in accordance with the Helsinki Declaration of 1975 [8].

All recruited subjects were ASA status 1 (healthy patients, according to the American Society of Anesthesiologists). The inclusion criteria were: age > 12 years, permanent dentition, no fixed appliance and no restorations on the occlusal surface of the assessed teeth. A total of 1011 permanent teeth (691 first molars and 320 second molars) in 255 patients, aged 13–20 years (mean age 16 ± 2.2 years), were evaluated. A preliminary 3-day theoretical and practical calibration course was conducted by an experienced dentist (LO), and the weighted kappa values were excellent both for ICDAS-II criteria, kappa = 0.87, and for VistaCam criteria, kappa = 0.92.

Two blinded and trained operators inspected the unrestored occlusal surfaces of each tooth with a plane dental mirror, in accordance with generally accepted standards [9]; the first assigned an ICDAS-II code while the second assessed the VistaCam score using a VistaCam iX Proof (Dürr Dental). The level of agreement between the two procedures was expressed using Cohen’s and Fleiss’ kappa statistics and performing the McNemar’s test. 

Prior to each diagnostic evaluation, a professional cleaning was performed with a rotary brush and a polishing paste in order to standardize the visual and fluorescence assessment of the occlusal surfaces. First, a trained dentist (D.C.) conducted a visual inspection to assess the ICDAS-II score. To standardize the conditions, all the diagnostic assessments were performed in the same dental unit, with the same source of light, a dental mirror and the use of compressed air, and in the morning. The ICDAS-II distinguishes six various stages of caries, along with a score of “0”, which means caries-free; from 1–3, which grades the alterations in enamel, to 4–6, which describes the alterations in dentin [2].

The new technology was then presented and explained to the patient and to parents or caregivers. Successively, the second blind operator (M.M.) assessed the VistaCam score.

The VistaCam iX Vista Proof Tip (Dürr Dental, Bietigheim-Bissingen, Germany) uses ultraviolet light of 405 nm length to locate and assess the severity of dental caries in the hard tissues of the tooth and distinguishes caries, tartar and plaque, displaying the results in a clear histogram applied to the image of the tooth surface on an attached computer screen [7,10]. The reflected light is filtered through light below 495 nm and contains green fluorescence with a peak at 510 nm, and red fluorescence of bacterial porphyrins with a peak at 680 nm. The received fluorescent beams are sent to the computer and processed by the software (DBSWIN, Dürr).

For subsequent evaluation, the Vista Proof values were classified as follows: 0–1.2 = sound tissue; 1.3–1.5 = enamel caries; >1.5 = dentine caries [5,10].

Table 1 shows the ICDAS-II scores and the corresponding fluorescence-based assessments.

## 3. Results

Some 283 (28%) of the assessed teeth were diagnosed as ICDAS-II code 0; 334 (33%) code 1; 189 (18.7%) code 2; 176 (17.4%) code 3; and 29 (2.9%) code 4. VistaCam assessed in 513 (50.7%) sound enamel (0–1.2); in 292 (28.9%) initial enamel decay (1.2–1.5); and in 206 (20.4%) dentine caries (1.5–3) (Table 2). 

The alterations diagnosed as ICDAS-II code 0 (*n* = 283) were classified by fluorescence camera as 244 sound, 32 enamel decay and 7 dentine caries. The teeth assessed as ICDAS-II code 1 were classified by fluorescence camera as 212 sound, 100 enamel caries and 22 dentine caries. Those evaluated as ICDAS-II code 2 were classified by fluorescence camera as 50 sound, 98 enamel caries and 41 dentine caries. A total of 205 occlusal surfaces were assessed as ICDAS-II codes 3 and 4, and were classified by VistaCam as sound in 3.4%, 27.7% enamel caries and 68.9% dentine caries (Table 2).

When assessing ICDAS-II codes 1, 2 and 3, there were 806 evaluated teeth. This sample was assessed with enamel caries in 28.5% (230) and with dentine caries in 8.7% (70). In total, 37.2% of the samples showed a false negative when using the ICDAS-II scoring system.

Table 3 shows the evaluation of treatment needs comparing the ICDAS-II test results by testing the same occlusal surfaces with a VistaCam fluorescence camera: 86.2% and 37% of the occlusal surfaces were assessed as sound by the fluorescence camera in the “no need for intervention” and in the “need for intervention” group, respectively (Table 3).

The first question to be answered was whether there was a systematic difference between the results obtained from each method. For a binary response, the data were assessed by performing McNemar’s test, a modification of the ordinary chi-square test that takes the paired nature of the responses into account. A statistically significant result (*p* < 0.05) shows that there is evidence of asystematic differences between the proportion of positive responses from the two methods. McNemar’s chi-square value was 142.15, when df = 1 with a *p*-value of <0.001. The weighted kappa test showed coefficients ranging from 0.553 to 0.641, with an estimate of 0.61. These results showed a poor agreement between the two methods. The adjusted rand index (ARI) was computed to further corroborate this point and an ARI = 0.25 was calculated, which is quite a low value.

## 4. Discussion

When a minimally invasive methodology is applied to dental routine on a daily basis, early caries assessment is of crucial importance to avoid under- and over-treatment. In this in vivo prospective clinical study, we investigated the diagnostic outcomes of caries detection using visual examination compared with a fluorescence-based camera on 1011 occlusal surfaces of first and second molar teeth. 

The null hypothesis to be tested was that the two diagnostic procedures would have the same outcome. The results of this in vivo study showed that significant differences existed when comparing the two procedures. Therefore, the initial hypothesis was dismissed.

ICDAS-II is a well-known clinical investigation protocol widely acknowledged by dentists worldwide. The ease of assimilation of its assumptions and criteria is appreciated. A recent study by Paraviainen et al. showed that third-year students, after the lecture, were able to apply the ICDAS-II protocol clinically without any problems with high specificity, accuracy and intra-examiner agreement. In accordance with the results of the current study, the authors showed that compliance with the histological examination was satisfactory only in more extensive dentinal cavities, leaving much to be desired in the case of initial caries. Moreover, the authors underlined that the use of auxiliary intra-oral tool increases the correctness of the diagnosis [11].

ICDAS-II criteria can not only cause problems for beginners, but also are often differently interpreted in everyday clinical practice by more experienced dentists. A very recent study evaluated the impact of ICDAS assessments of occlusal caries in a high-risk population on the clinicians’ treatment decisions. Interestingly, the strongest correlation for inter-examiner reproducibility was found for lesions of ICDAS ≥ 3, whereas an inconsistency was seen for initial caries, according to ICDAS ≤ 2. The authors suggested that these inaccuracies, especially in patients with a high risk of caries, can often lead to unnecessary over-treatment, which blurs the currently accepted philosophy of minimally invasive dentistry [12]. This statement was confirmed by the other exam, while checking whether the diagnoses made using the visual–tactile method, according to the ICDAS protocol, correlated with the histological results of the examined changes. The biggest errors with diagnoses made by the experienced dentist were found in the changes consistent with codes 1 and 2 [13]. Moreover, in accordance with the results of the current study, the fluorescence-based methods showed greater internal and external validity than the subjective procedures, such as visual and tactile, in diagnosing occlusal caries in permanent teeth [6].

These results agree with our observations. Indications from VistaCam suggest completely different diagnoses in cases of alterations located in the enamel (ICDAS-II codes 1 and 2) (Table 2), and, as in the study of Qudeimat et al., only use of ICDAS protocol without any auxiliary tools can lead to over-treatment [12]. One-third of the examined surfaces in the present study was considered by software as sound tissue, without any need for treatment (Table 3). Moreover, our results showed that ICDAS-II underestimates 37.2% of enamel and dentine caries when assessing the occlusal surfaces as code 0–1–2, with low diagnostic sensitivity and specificity.

Jablonski-Momeni et al. evaluated the performance of ICDAS-II, laser fluorescence, fluorescence-based camera, and radiographic examinations for occlusal caries detection in order to assess their influence on treatment decisions when used as a single diagnostic method or as a combination of two methods. Their results showed that the use of the fluorescence-based camera is the most accurate evaluation method for assessing the condition of the dental surface before the establishment of a treatment plan, followed by ICDAS-II and other methods of evaluation [7]. In addition, in the case of uncertainty for the need for treatment, VistaCam remains a great help in monitoring the development of carious lesions in pits and fissures, even over a longer period of time [14,15,16].

Moreover, VistaCam showed efficacy in evaluating the residual carious tissue, appearing to be a useful supplementary tool in assessing the endpoint of caries excavation [17]. In addition, VistaCam was shown, in a recent in vivo study, to be effective in assessing and monitoring enamel demineralization under a clear sealant over a 12-month period of follow-up [15]. No data are available on VistaCam use in detecting secondary caries under filling restorative materials; in these cases, radiographic examination and laser fluorescence are the best diagnostic standards [18,19]. No data are present in the literature about the usefulness of a fluorescence-based method when applied to the new scaffold and biomimetic materials [20,21].

When taking into account the aspect of practical application during epidemiological studies, it is important to remember that ICDAS-II ensures detailed information about lesion severity, but it is considered as a time-consuming and hard to analyze method, also in the conditions of the dental office. Moreover, out of the office, it is even more difficult to keep the mouth dry and to ensure standardized and sufficient lighting conditions, which makes it impossible to carry out epidemiological studies and correct diagnosis of early carious lesions [22].

The use of VistaCam also presents some limitations, as examinations should be carried out under certain conditions. The functioning intra-oral camera should not be disturbed by the presence of saliva on the tested hard tissues, and blood should be removed before the test, because it contains porphyrin derivatives, such as hemoglobin. The presence of these compounds enhances the fluorescence and increases the value of results [17,23]. In addition, a previous study has shown that the VistaCam assessments should be carried out after the removal of plaque from occlusal surfaces [24].

### Limitations

The limitations of this study are principally due to the availability of the proposed diagnostic technology. The use of a fluorescence-based camera assumes that it is purchased, and on a global scale there are realities where dentistry has yet to be based on the possibility of finding the basic means of assistance. That is to say that ICDAS-II remains a more democratically available method for caries screening, with unfortunately a low sensitivity for early caries detection. On the other hand, the strengths of the clinical study reported here are mainly based on a new diagnostic, non-invasive and not operator-sensitive technology that permits one to safely and continuously monitor the enamel demineralization at early stages.

## 5. Conclusions

In conclusion, the data from the current in vivo prospective clinical study suggest that there is poor agreement between the two diagnostic methods, especially in ICDAS-II codes 0, 1 and 2 and fluorescence evaluations. Future randomized studies will better define the role of the operator’s visual inspection and the fluorescence-based camera. Until then, usage of ICDAS-II and fluorescence-based cameras together should continue to be considered the standard of care and begin to be widely used in daily dental routine. 

## Figures and Tables

**Table 1:** Description of visual and fluorescence-based diagnostic criteria.

Visual Caries Detection (ICDAS-II)	VistaCam Fluorescence-Based Camera
0 = Surface not restored or sealed	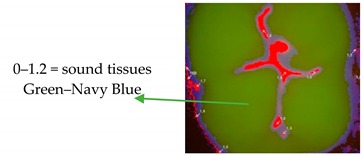
1 = First visual change in enamel: Opacity or discoloration (white or brown) is visible at the entrance to the pit or fissure seen after prolonged air drying. 2 = Distinct visual change in enamel visible when wet, lesion must be visible when dry. 3 = Localized enamel breakdown (without visual clinical signs of dentinal involvement) seen when wet and after prolonged drying.	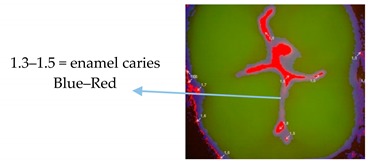
4 = Underlying dark shadow from dentine 5 = Distinct cavity with visible dentine 6 = Extensive (more than half the surface) distinct cavity with visible dentine	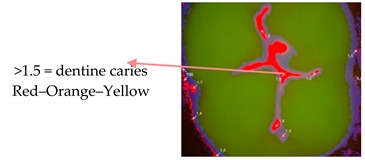

**Table 2:** Evaluation of ICDAS-II test results by testing the same occlusal surfaces with a fluorescence camera (VistaCam Proof).

Visual–Tactile Examination (ICDAS-II)	No. of Teeth	The Results from VistaCam Examination		
		SOUND	ENAMEL CARIES	DENTINE CARIES
CODE: 0	283	244 (86.2%)	32 (11.3%)	7 (2.5%)
CODE: 1	334	212 (63.5%)	100 (29.9%)	22 (6.6%)
CODE: 2	189	50 (26.5%)	98 (51.9%)	41 (21.6%)
CODE: 3	176	6 (3.4%)	55 (31.3%)	115 (65.3%)
CODE: 4	29	1 (3.4%)	7 (24.1%)	21 (72.5%)

**Table 3:** Evaluation of treatment needs comparing the ICDAS-II test results by testing the same occlusal surfaces with a VistaCam fluorescence camera.

Visual–Tactile Examination (ICDAS-II)	No. of Teeth	The Results from VistaCam Examination		
		SOUND	ENAMEL CARIES	DENTINE CARIES
CODE: 0 (no need of intervention)	283	244 (86.2%)	32 (11.3%)	7 (2.5%)
CODE: >1 (need of intervention)	728	269 (37%)	260 (35.7%)	199 (27.3%)
